# The Influences That Diseases of the Mouth, Nose and Throat Have upon Individual and Community Health

**Published:** 1912-06-15

**Authors:** Horace W. Starkey

**Affiliations:** Rockford, Ills.


### THE INFLUENCES THAT DISEASES OF THE MOUTH, NOSE AND THROAT HAVE UPON INDIVIDUAL AND COMMUNITY HEALTH.

BY HORACE W. STARKEY MD., ROCKFORD, ILLS.

Abstracted from an Article Printed in the *Dental Review.*

### THE NOSE AND THE THROAT.

The nasal spaces are, in health, essentially two tubes with irregular walls extending from the face to the throat, and connected with the various sinuses in the surrounding bones, with the lachrymal canal, and with the ear. Each space is formed, in a general way, by a floor which is level so that secretions may readily find their way forward or backward according to the position of the head; of an inner wall or septum which should be perpendicular, straight and smooth, so as not to interfere with the free passage of air; an outer wall, very irregular from the projection of the turbinate bodies, and a roof or vault which is even more irregular and which contains most of the openings to sinuses. This whole space is covered with mucous membrane which is continuous with that of the alimentary and respiratory tract, with the conjunctiva through the lachrymal canal, with the drum of the ear through the Eustachian tube, and with the sinuses.

The principal functions of the nasal spaces are: respiratory, olfactory, gustatory and phonatory. While the latter three are, as we shall see, very important, the respiratory function is the most important—so important in many of the lower animals and in infants as to be vital, as life can not be preserved without respiration through the nose—and where not vital it is of extreme importance because of the changes wrought in the air in its passage through the nose.

The variations in the air in a climate and surroundings such as we are exposed to are extreme and often very sudden. The air may be very moist, or it may be very dry; it may have a very high temperature or it may be very cold; it may be pure and contain the proper proportion of ingredients, or it may be foul from dust, smoke, soot and other impurities and may carry very many noxious micro-organisms.

It is one of the duties of the nose to receive air in all these different conditions and so change it that it shall pass into the lungs in an approximately uniform condition at all times. The mucous membrane of the nose is very rich in blood vessels which convey heat and moisture, and this is particularly true of the erectile tissue or swell bodies which are located mainly at the posterior ends of the lower and middle turbinates, and along the lower border of the lower turbinates When the air is dry or cold these bodies enlarge, and so present a larger surface to warm and moisten it, and when it is warm and humid they contract under the control of regulating nerves. It is estimated that in dry weather a pint of water daily is evaporated from the nose by the inspired air.

In addition to warming and moistening the air, the nose also filters from it dust, smoke, soot, pollen of flowers, etc., and also catches and, to a certain extent, destroys the various micro-organisms which constantly teem in the air—

thus saving the very delicate structures lower down in the respiratory tract from the deleterious effects of these foreign and irritating particles.

That the olfactory sense is important we see when we consider the pleasure we derive from agreeable odors and also the promptness with which this sense warns us of noxious vapors, gases and emanations. One who has not an acute sense of smell is in much more danger from all of these than one who has.

The gustatory function is only slightly inferior in importance to the olfactory. We may not appreciate the fact that very much of one's enjoyment of food is due to the nose. We taste sweet, sour and bitter, but the delicate flavors and aromas are perceived by the nose.

The last function of the nose is phonation. The nasal passages act as a sound-box or resonator to the mouth and throat in vocalization, just as certain parts of musical instruments are added for that purpose. If the nasal passages are of proper capacity and shape, the voice may be clear, pure-toned and sweet, but if there is much obstruction of these passages the voice is thick and muffled, while with too much space and without proper checks upon the air, such as are normally provided by the turbinate bodies, the voice will be harsh and strident.

### VALUE OF THE TONSILS.

Just where the nose and mouth come together are three very important structures, the three tonsils; one on each side of the pharynx partly below and partly above the soft palate, and the third on the back of the epipharynx just above the soft palate. The latter is ordinarily called adenoid or, less correctly, adenoids. These three are all glands of similar structure and are very active in infancy and early childhood, then they usually atrophy and are very small during the rest of life. Their function is not well known, but their location, forming a ring about the portal to the lungs and stomach, and their activity in infancy, indicates that they have some important work. And their subsequent atrophy would indicate that this work was done, but we do not know that the remnant of tonsils does not continue useful; and the discoveries of the internal secretions of glands, made in the last few years would point to a large possibility of such usefulness. Recently R. H. Good, *Laryngoscope*, June, 1909, and B. D. Sheedy, *Medical Record*, September 25, 1909, have, at about the same time advanced a theory that seems rather plausible, and which may be tentatively received for further investigation. Dr. Good and Dr. Sheedy believe that the tonsils have to do with immunization. Very great advances have been made in our knowledge of immunity in recent years. It has been known for centuries that the occurrence of certain diseases in individuals, rendered those individuals immune, or not susceptible, to a second attack, in many diseases for the rest of life, in others for a limited time.

We now know in a general way that the presence of the micro-organisms and toxins of certain diseases in the system stimulate the blood to produce anti-bodies and anti-toxins that destroy those microbes and toxins, and also stimulate the white blood cells to destroy such micro-organisms as produce these diseases. In certain diseases this added quality of the blood is permanent, in others, temporary. And we also have found that the repeated exposure to relatively small numbers of certain microbes, such small numbers that no untoward effect is produced, has the same or even a more certain effect in producing immunity as the one great exposure in the case of such a disease as scarlet fever or smallpox. With every breath and with every mouthful of food, we take in numberless micro-organisms, many of them harmful to the unimmunized individual, more of them indifferent, and perhaps most of all beneficial, and we all constantly have great numbers of various virulent microbes such as those producing tuberculosis, pneumonia and other septic conditions in our noses and mouths. But to these, in the numbers ordinarily present, we are immune when in robust health, and this immunity must be acquired. And the theory mentioned above, credits the tonsils, the three tonsils, mind you, with being an important factor in this immunity. These forming a ring about the naso-pharynx are so situated that both air and food must pass over them. And, as in infancy, they present a relatively large surface and have a very active circulation, they are in the most favorable possible position for receiving, and disposing of large numbers of these little foes, and thus are able to render the individual immune to the ordinary micro-organisms, and having done their work, nature allows them to become quiescent. At any rate, the tonsils must perform some important service or they would not be so sedulously cared for in the economy. Whether in their diminished activity they still perform some useful function in adults, is wholly unknown.

Normally the nasal spaces are sufficiently free so that enough air can pass through on either side alone, to meet the ordinary needs of respiration, the throat is so patulous that it presents no obstruction at all, and the larynx while permitting free respiration, also performs easily and unconsciously all the complicated movements required in phonation and vocalization; and the mouth receives the food and prepares it by mastication, insalivation, warming or cooling it, as may be required, and passes it on to the esophagus, the soft palate, and the epiglottis, at the same time closing the air spaces above and below.

### DISEASES OF THE NOSE.

Diseases of the nose may be divided broadly according to their influence upon the individual, into two groups: those that cause obstruction and those that cause undue enlargement of the nasal spaces. The former are altogether the most numerous, but the latter, while less frequent, are much more difficult to relieve, and produce very great discomfort and very serious consequences. In most cases of obstruction, we can increase the patency of the space and relieve the accompanying symptoms and discomforts. But when the turbinate bodies are dimished in size and the mucous membrane, and particularly that part containing the swell bodies, is destroyed, there is little that we can do, and the individual must go through life with all the discomforts and dangers of the condition.

The causes of obstructive nasal disease are traumatic, climatic and septic. Traumatic, from falls, blows, etc. When we see the way in which young children fall on their noses, we wonder that any of them ever grow up with the nose in proper shape, but few falls result in any real damage to the nose. But the possibility of such damage should always be kept in mind and immediate attention given to replacing broken bones and dislocated parts. When this is neglected, much more serious operations must, in many cases, be undertaken in after years.

The climatic conditions in this latitude are such as to predispose to colds and catarrh, which are likely to lead to obstruction through swelling and thickening of the mucous and submucous tissues. The tendency to colds must be combated by hygienic measures, such as proper clothing and diet, pure air, cool baths, and can frequently be much diminished by the use of bacterial vaccines to improve the condition of the blood so that it will destroy the microorganisms usually producing colds.

Obstruction of the nose interferes with free respiration, and the air not being sufficiently warmed, moistened and filtered, causes irritation and ultimate disease in the delicate structures of the air cells and other tissues. Insufficient air entering the lungs, the blood is imperfectly aerated, and the individual suffers from insufficient oxidation and from excess of carbonic acid in the blood. In children, the nose, not being used normally, collapses, and, the mouth being kept open for breathing, the jaw bones do not develop properly, and we have the characteristic expression, the adenoid face; and the lungs being imperfectly expanded, we get the contracted chest. Moreover, this condition of the nose is likely to effect some or all of the surrounding structures, particularly those to which the nasal mucous membrane extends. Most cases of lachrymal disease with overflow of tears are caused, or complicated by nasal disease, and we see many cases of persistent conjunctivitis and severe irritation of the eyes, that will not recover until the nasal disease upon which they depend is relieved. Through obstruction of their outlets or through direct extension of inflammation, any of the connecting air spaces may become involved causing painful and dangerous disease in the sinuses—dangerous because it may extend to the brain, causing death. Also, we have been finding out in the past few years that some of the most insidious and hitherto unexplained diseases of the interior of the eye, diseases which frequently lead to total blindness, are caused by sinus infection, and promptly disappear when the sinus trouble is cured.

Most all acute and chronic ear trouble is due to nasopharyngeal disease. Through the Eustachian tube the disease passes to the ear, and there causes pain, deafness, noises in the ear, discharge from the ear, mastoid disease, with sometimes fatal effects from septicemia or brain disease.

On the other hand there are diseases that cause atrophy of the nasal structures, enlarging the nasal passages, and destruction of the mucous membrane, so that air goes too freely into the lungs without sufficient warmth, moisture or filtering. These patients are very uncomfortable, from a feeling that the air passes through the nose too freely; crusts form from dryness with irritation of the nose, throat and the lungs. These also frequently lead to disease of surrounding parts, but not so frequently as the obstructive variety. Fortunately, these cases of idiopathic atrophic rhinitis are rather rare, but unfortunately we see many traumatic cases, due to the removal of too much tissue in operations that are not so rare.

### DISEASED TONSIL.

In the throat, the most important condition from the dentist's point of view, is diseased and enlarged pharyngeal tonsils. While you may frequently see other diseased conditions, such as follicular pharyngitis, ulcerations, enlargements and diseased blood vessels, to which the attention of the patient may be called, the tonsil cases are altogether more numerous than all others, and the careful dentist will call attention to this condition, as well as to nasal diseases, and advise that it receive efficient treatment.

While normally the tonsils cease their great activity in early childhood, and shrink to inconsiderable masses, in frequent instances this does not occur, but one or all remain large or even increase in size and become diseased, and cause much trouble. This enlargement may cause obstruction to respiration, with all the symptoms and dangers mentioned above in nasal obstruction, and their disease may be accompanied by pockets in which food is caught and undergoes decomposition, and in which the micro-organisms, that are normally destroyed, find a most congenial place for multiplying and pass from here to the blood and lymph channels. This absorption of microbes and their toxins is one of the most frequent, if not the most frequent of causes of tuberculosis of the glands of the neck, and of some systemic diseases. We now know, for instance, that very many cases of acute and chronic rheumatism are due to diseased tonsils.

Besides the means used to promote general good health, local measures may be adopted to relieve or cure the conditions present. These are surgical or operative, and topical, as with applications of sprays, vapors, lotions, ointments, etc. There are many who think and teach that the only beneficial treatment of throat and nose disease is operative, and that one is throwing his time away in treating it otherwise. With this contention I have no sympathy. Many patients are relieved and kept comfortable and well for years by applications to the effected tissues without operative interference. On the other hand there are some in which operation offers the only hope of relief, and here the patient should receive the benefit of prompt, careful and skilful surgery. But no operation should be undertaken without a definite idea as to what it is expected to accomplish, and also definite knowledge as to the results that should follow the operation—not only the immediate but the remote results. An operation should only be undertaken when there are definite indications for it, and then it should be so devised as to meet those indications with the least possible destruction of tissue, and no destruction of useful tissue unless it is absolutely necessary, always remembering that it is easier to remove parts than to replace them.

There has been in the past, and is now, too much reckless surgery in the nose. With too many, when a patient with some obstruction to the nasal spaces presents himself the first and only thought is to "clean out the nose," and in doing this parts are sacrificed that should be preserved, and the last state of that patient is worse than the first. The immediate results may be excellent and the patient and physician congratulate themselves on a most happy cure, but in a few weeks the changes commence that result in the enlarged nasal spaces, and never again is that patient either well or comfortable. We see altogether too many such patients. The indications for operation in the nose are to improve drainage and secure easy respiration, and if these can be met with little or no destruction of tissue the greatest possible good with the least possible harm has been secured. In particular should the lower border and ends of the inferior turbinates be avoided, for here we have found that the swell bodies are principally located.

And what is true of the nose is also true of the nasopharynx. Here the three tonsils are the principal offenders, and the principal objects of surgical interference. But there are just three indications for the removal of these bodies. The first of these is obstruction. When the tonsils, instead of undergoing contraction in early childhood, become enlarged and by obstruction interfere with respiration, they, or at least the diseased portion, should be removed.

A second indication is infection. When the more or less enlarged tonsil is full of crypts and pockets that are, or may become, culture tubes for micro-organisms, thus endangering or infecting other parts, or the whole body, it should be removed and this source of danger eliminated. And when tonsil or adenoid is the frequent seat of inflammation, and the individual is sick with tonsilitis or quinsy, the offending member should be removed. But the mere persistence of some tonsillar or adenoid tissue which can be seen or felt but which is flat and smooth and has no crypts and is not subject to inflammation, is not an indication for surgical interference. Such a flat mass is doing no harm, and may be doing some good, so it should not be interfered with.

But when an operation is necessary it should be performed by an expert. There seems a vicious idea prevalent among physicians and the laity that anyone can perform a tonsil or adenoid operation, and we find men everywhere, without special training or skill, undertaking these operations and doing them in a most slovenly and imperfect manner.

After a very long experience, I would say that I do not consider a tonsil-adenoid operation either simple, easy or devoid of danger, but between this and a simple appendicitis operation the latter is less severe and requires less experience and skill. My plea is that those who wish to do work upon the nose and throat, as well as those who take care of the teeth, and the eyes, should be willing to devote enough time and care to preparation to be able to do that work in a proper manner. Because a tooth aches, you are not to extract it, or because a nose is obstructed we should not remove the turbinates, or because there is some tonsillar tissue found to go after it.

COMMUNITY HEALTH.

The community's health is the total of the health of the various separate individuals that compose that community, and that which causes the sickness and death of an individual, by so much lessens the total efficiency of the community. But in addition to this, if the sickness of the individual is contagious and, so, likely to extend to and incapacitate others, by so much more it becomes a matter of general concern. While diseases of the mouth, nose and throat directly cause but a very small proportion of deaths, they indirectly cause a much larger proportion, and they do cause a very large part of the common disability from sickness. I think we shall appreciate this the more if we each of us think of the sickness that has occurred in our own families and among our immediate friends in, say, the past five years. Ordinary colds and sore throats of all kinds, practically all ear troubles, most cases of grippe, and all cases of sinus diseases would at once occur to all of you as being included, but, in addition, most cases of diphtheria, scarlet fever, measles, smallpox and other contagious diseases would come under this head because all such infectious diseases find their means of entrance to the body through the nose and mouth.

Of equal importance to the individual and to the community is the influence that disease of the naso-pharynx has upon the susceptibility of the individual to all other diseases. As representing all, we will consider the condition of a child having, we will say, enlarged and diseased tonsils and adenoid. Such a child has difficulty in breathing so that the nervous and muscular systems are constantly overtaxed and the child is constantly tired, this weariness being increased by a lack of a proper amount of restful sleep. Respiration being inadequate, there is lack of proper aeration of the blood, which, in turn, affects digestion, and assimilation and nutrition is below par. All of this in turn reacts upon the blood so that its resisting and bactericidal powers are diminished and the child is an easy victim of any contagion to which he may be exposed. The strong, hearty child may be exposed to a considerable contagion and not contract disease, while the child we have just described succumbs to a much less exposure, and sickness of any kind is much more severe, more prolonged and more dangerous than in the robust.

Thus is the total amount of sickness and mortality in the community increased, but the influence does not cease here. This child having contracted disease, is liable to impart it to others, and so the circle grows, not only in children, but in adults, for the adult with poor nutrition and unstable nervous system is also much more susceptible to disease, and among such subjects pneumonia, tuberculosis and all contagious and infectious diseases find a very large proportion of their victims; and he is more easily a victim, not only because his general health is impaired, but also because the nose and throat presents a ready place of entrance for the micro-organisms producing these various diseases.

Each day's sickness and each life lost is an economic loss to the community, for we must remember that not only in adults is sickness an economic loss, because of time taken from a useful occupation, but in children also lost time means loss of schooling, and as the community pays for the schools whether the child is present or absent, each day's absence of a pupil means direct pecuniary loss for which the taxpayer must pay; and when we add to the loss in time of those sick, and the loss to the community of the value of the lives of those who die; the loss in efficiency of those who continue to struggle on with energy impaired by the diseases we are discussing; of those totally or partially blind from this cause, and of those totally or partially deaf from this cause, we see how great a factor in the individual welfare and in the welfare of the community the mouth, nose and throat is. In addition we must remember that the physical defects so caused are but part of the story and that accompanying this are mental deficiencies and moral obliquities, for it is well known that the mind is less active and acute when the general physical condition is below normal, and also that many children with perverted moral tendencies become normal when physical defects are removed. We see now how great a problem we are discussing, and realize the necessity for a general awakening of the community to the economic waste of poor physical conditions. We are convinced that attention should be called far and wide to the important role of diseases of the mouth, nose and throat.—*Dental Review.*

